# The Association Between Low Socioeconomic Status With High Physical Limitations and Low Illness Self‐Perception in Patients With Juvenile Idiopathic Arthritis: Results From the Childhood Arthritis Prospective Study†, ‡

**DOI:** 10.1002/acr.22466

**Published:** 2015-02-24

**Authors:** Suzanne M. M. Verstappen, Joanna Cobb, Helen E. Foster, Bo Fu, Eileen Baildam, Lucy R. Wedderburn, Joyce E. Davidson, John Ioannou, Alice Chieng, Kimme L. Hyrich, Wendy Thomson

**Affiliations:** ^1^ Arthritis Research UK Centre for Epidemiology, Centre for Musculoskeletal Research, Institute of Inflammation and Repair, The University of Manchester, Manchester Academic Health Science CentreManchesterUK; ^2^ Newcastle University and Great North Children's Hospital, Newcastle Hospitals NHS Foundation TrustNewcastle upon TyneUK; ^3^ Arthritis Research UK Centre for Epidemiology, Centre for Musculoskeletal Research, Institute of Inflammation and Repair, Manchester Academic Health Science Centre, and Institute of Population Health, The University of ManchesterManchesterUK; ^4^ Alder Hey Children's Foundation NHS Trust, HospitalLiverpoolUK; ^5^ UCL Institute of Child Health, Arthritis Research UK Centre for Adolescent Rheumatology at UCL, UCLH and GOSH, and Great Ormond Street HospitalLondonUK; ^6^ Royal Hospital for Sick Children, Greater Glasgow and Clyde Health Board, Glasgow, and Royal Hospital for Sick ChildrenEdinburghUK; ^7^ Arthritis Research UK Centre for Adolescent Rheumatology, University College LondonLondonUK; ^8^ The Royal Manchester Children's HospitalManchesterUK; ^9^ Arthritis Research UK Centre for Epidemiology, Centre for Musculoskeletal Research, Institute of Inflammation and Repair, The University of Manchester, NIHR Manchester Musculoskeletal Biomedical Research Unit, Central Manchester NHS Foundation Trust, Manchester Academic Health Sciences CentreManchesterUK

## Abstract

**Objective:**

To examine the association between socioeconomic status (SES) and delay to a pediatric rheumatology clinic, disease severity, and illness perception in patients with juvenile idiopathic arthritis in England.

**Methods:**

Using the Index of Multiple Deprivation, 923 consecutive children from the Childhood Arthritis Prospective Study were assigned to SES groups: high‐SES (19.1%), middle‐SES (44.5%), or low‐SES (36.4%). At baseline, disease activity was assessed, and the Childhood Health Assessment Questionnaire (C‐HAQ), the Illness Perception Questionnaire, and the Child Health Questionnaire, version Parent Form 50, were completed. Linear median regression analyses or zero‐inflated negative binominal (ZINB) regression analyses were used.

**Results:**

Delay to first pediatric rheumatology consultation was the same between the 3 SES groups. Although disease activity scores assessed by the pediatric rheumatologist did not differ between the 3 SES groups, persons in the low‐SES group recorded higher C‐HAQ scores compared to the high‐SES group (zero‐inflated part of ZINB odds ratio 0.28 [95% confidence interval (95% CI) 0.14, 0.55], count part of ZINB β 0.26 [95% CI 0.05, 0.48]). Parents with low SES also reported more often that their children's school work or activities with friends had been limited. Furthermore, the low‐SES group had a worse perception about the consequences of the disease and the effect of treatment than those in the high‐SES group.

**Conclusion:**

Patients from a low‐SES background report more problems with daily activities and have a lower perception of the consequences of the disease than patients from a high‐SES background, warranting special attention from a multidisciplinary team.

## INTRODUCTION

Juvenile idiopathic arthritis (JIA) is a chronic inflammatory disease of childhood with an incidence in the UK estimated to be 10 per 100,000 1. JIA is a heterogeneous disease, and during childhood and adolescence many patients experience physical and psychosocial limitations, including functional disability, pain, absence from school, and difficulties participating in other activities such as sports 2, 3. JIA not only causes problems during childhood and adolescence, but joint damage accrued during these years and ongoing exacerbations of inflammation are associated with functional and socioeconomic consequences into adulthood 2, 4, 5. With respect to the latter, a few studies have shown that despite higher rates of school absence, patients with JIA have similar or above average high school grades compared to the general population in the UK, but patients with JIA are less likely to be employed after leaving school 6, 7, 8.

Socioeconomic status (SES) may not only be a consequence of poor health, but may also influence many aspects of health and well‐being 9, 10. Research has shown inequity due to differences in SES regarding access to health care systems and drugs, across countries and within countries 11. SES is often measured at an individual level based on formal education, income, social class, or occupation. In addition to differences in individual backgrounds, people are also affected by their environment and the opportunities to live in certain areas (i.e., deprivation at an area level). Although these concepts of measuring SES differ slightly, they have both shown to exhibit independent effects on outcome in arthritis and are used interchangeably, and thus are both referred to as SES in this article 12. In populations including patients with adult rheumatoid arthritis, those from deprived areas have more severe disease activity early in the course of the disease and have worse disease progression over time 13, 14, 15, but conflicting results have been found with respect to access to health care 16. Little is known in JIA about the impact of SES on disease severity, illness perception, and delay to pediatric rheumatology at presentation. In 1 US study of 295 patients with JIA, the patients on Medicaid, a health care program for those with low income and resources, had higher disability and lower health‐related quality of life compared with privately insured JIA patients, suggesting an adverse impact of low SES 17. Both groups of patients had similar access to care, with the exception of magnetic resonance imaging (MRI), because privately insured patients were almost twice as likely to receive MRI scans, giving the chance of tighter disease control. Early and tight disease control is essential, and delay in referral may have a profound effect on long‐term outcomes such as joint damage and functional disability. Parents with higher education are more likely to see a pediatric rheumatologist early than parents with minimal or no education 18. Controversial results have been found when looking at the association between distance from a patient's home to the clinic as a proxy for SES in referral to a pediatric rheumatologist 18, 19. The results from these studies suggest that factors associated with SES may play a role in disease outcomes. Although no definitive measure exists, by directly investigating SES via more accurate measures such as area‐specific deprivation scores, in addition to more detailed data on outcomes, we could gain a clearer understanding of the impact of SES on disease related variables at presentation and delay to access in patients with JIA.

The aims of the present study were to investigate the impact of SES on disease severity early in the disease, on delay in accessing pediatric rheumatology care, and on illness perception in the Childhood Arthritis Prospective Study (CAPS), a large observational cohort of patients with JIA in the UK followed over time.

Box 1 **Significance & Innovations**

- Parents of patients with juvenile idiopathic arthritis (JIA) or children with JIA from a lower socioeconomic background report more functional disability, while disease activity according to the pediatric rheumatologist is the same.
- Parents of patients with JIA from a lower socioeconomic background more often report that their children's school work or activities with friends had been limited due to emotional difficulties or problems with their behavior.
- In this cross‐sectional study, parents of patients with JIA or children with JIA have different perceptions regarding the burden of the disease, depending on socioeconomic background, warranting special attention from a multidisciplinary team over a longer period of time to understand the causal relationship of this finding.

## PATIENTS AND METHODS

Participants were children (ages <16 years) with inflammatory arthritis of at least 1 joint persisting for ≥2 weeks who were recruited to the study. CAPS is a multicenter study in the UK with an overall aim to identify genetic and environmental predictors of short‐term and long‐term outcomes of inflammatory arthritis. Details of this study have been described elsewhere 4, 20. CAPS was approved by the North‐West Multi‐Centre Research Ethics Committee. Written informed consent was obtained from the parent(s)/guardian for all participating children according to the Declaration of Helsinki, and children considered able were asked to provide age‐appropriate assent.

### Baseline data collection

At baseline (the first visit to a pediatric rheumatologist), the rheumatologist examined the joints, recording the number of limited and active joints (out of 71 joints), completed a 100‐mm physician's global visual analog scale (VAS; range 0–100 mm, where 100 is the worst score) and assigned an International League Against Rheumatism (ILAR) subtype of JIA based on the disease characteristics at the time of presentation 21. In addition, the parents and child were interviewed by a rheumatology research nurse, and the medical records were reviewed to extract data on demographics (age, sex, diagnosis, source of referral, disease duration, past medical history, medication use, and relevant blood tests, such as erythrocyte sedimentation rate [ESR]). The parent or child, where appropriate, completed the British version of the validated Childhood Health Assessment Questionnaire (C‐HAQ), a measure of functional disability (range 0–3, where 3 is the worst) 22, a 100‐mm VAS pain scale, and a 100‐mm parent/patient global measure. The C‐HAQ was completed by the parent if the child was ≤10 years or by the child if age ≥11 years. The active joint count, physician's global assessment, ESR, and parent/patient global assessment were used to calculate the Juvenile Arthritis Disease Activity Score based on 71 joint count (JADAS‐71) and the JADAS3‐71 (which excludes ESR) 23, as a measure of disease activity.

To evaluate the perception of how a child's arthritis affects their life, the Illness Perception Questionnaire (IPQ) 24 was also completed by the parent (child age ≤10 years) or by the child (age ≥11 years). In this study we only included section 2 of the IPQ, evaluating the personal views about JIA. Answers were rated on a 5‐point Likert scale, ranging from “strongly disagree” to “strongly agree.” The Child Health Questionnaire (CHQ), in the version Parent Form 50 (CHQ‐PF50) 25 was completed by the parents of children ages ≥5 years (see Supplementary Appendix A, available in the online version of this article at http://onlinelibrary.wiley.com/doi/10.1002/acr.22466/abstract, for a description of the IPQ and CHQ‐PF50 items included in the current study). To aid interpretation of the CHQ‐PF50 scores, scores were reversed in the current study (i.e., higher score = worse score). Both the IPQ (from September 2003) and the CHQ (from December 2001) were introduced a few years after the start of CAPS, and therefore fewer parents/children completed these forms.

### SES

For the present study, we used postal codes to assign each patient to a nationwide deprivation rank by using the most recent nationwide Index of Multiple Deprivation (IMD) score compiled in 2007 (http://geoconvert.mimas.ac.uk/). We then assigned patients to nationally determined quartiles of deprivation (the lowest quartile = least deprived group [high‐SES], the middle 2 quartiles = middle‐SES group, and the highest quartile = most deprived SES group [low‐SES]). The IMD is a measure of multiple deprivations at the small‐area level. It breaks the areas down to the lower super output area level (a minimum population area of 1,000 people). These levels are a constant size across England and not subject to the regular boundary changes of electoral wards, and they combine indicators from economic, social, and housing issues into a single deprivation score. Seven separate weighted domain scores were combined to make the IMD, including income, employment, health deprivation, disability, education, skills, training, barriers to housing and services, crime, and living environment. Only children recruited in England were included in the present study, since the IMD is only based on data collected in England.

### Statistical analysis

Descriptive data, including demographics, disease characteristics (JADAS3‐71, JADAS‐71, active and limited joints, physician global, parent global, and C‐HAQ scores), IPQ item scores, and CHQ‐PF50 item scores, were presented for the total study population and divided by SES group (low‐SES, middle‐SES, and high‐SES groups). Logistic regression analyses were used for binary outcomes. For continuous variables, the association between SES and baseline characteristics was investigated by applying univariate linear regression analysis, median regression analysis, or zero‐inflated negative binominal (ZINB) regression analysis, depending on the distribution of the data. Median regression is a robust alternative to the usual linear (mean) regression when the linear regression assumptions, mainly the assumption of normality of the residuals, is violated due to skewed distributions for response variables 26. ZINB regression analysis allows for an excessive number of outcome zeros by assuming that zero outcome is due to 2 different processes. First, a logit model is generated to predict whether a child belongs to the certain‐zero group. Second, a negative‐binominal model is generated to predict the counts for those children who are not certain zeros 27. The Vuong statistic was considered for the zero‐inflated model to determine whether it is an improvement over the standard negative‐binomial model. Beta coefficients were transformed into odds ratios (ORs) for the zero‐inflated part. The C‐HAQ score was one of the outcomes for which ZINB regression analysis was applied, and the C‐HAQ score (0.125 incremental scale) was therefore multiplied by 8 to obtain counts. A multinominal logistic regression analysis was applied to assess the association between SES and point of referral. All analyses were undertaken using STATA software, version 11.0.

## RESULTS

A total of 923 children with a median age at symptom onset of 6.7 years (95% confidence interval [95% CI] 2.8, 10.7) for whom the IMD rank could be determined were included in the study (Table 1). The majority of patients (89%) were from a white cultural background, 4% were from a mixed background, and 7% were from Asian, black, or other background. The median C‐HAQ score was 0.625 (95% CI 0.125, 1.375), and the recorded CHQ‐PF50 scores of all items were higher than previously seen in healthy children 25. The mean ± SE original score for physical function was 61 ± 1.8 versus 96 ± 0.8, and for role/social limitations–emotional/behaviorial 73.5 ± 1.8 versus 92.9 ± 0.9, healthy children versus JIA patients, respectively.

**Table 1:** Baseline demographic and disease characteristics*

	N	Value
Age at onset, years	923	6.7 [2.8–10.7]
Female, n (%)	923	581 (63)
Delay to rheumatologist, months	920	5.6 [2.9–12.0]
Active joint count	905	2 [1–4]
Limited joint count	905	1 [0–3]
Physician global assessment, mm	713	29 [15–51]
ILAR subtype, n (%)	842	
Systemic arthritis		48 (5.7)
Persistent oligoarthritis		430 (51.1)
Extended oligoarthritis		18 (2.1)
RF‐negative polyarthritis		145 (17.2)
RF‐positive polyarthritis		29 (3.4)
Enthesitis‐related arthritis		44 (5.2)
Psoriatic arthritis		44 (5.2)
Unclassifiable		50 (5.9)
Other inflammatory arthritis		34 (4.0)
JADAS‐71	304	11.2 [5.8–17.2]
JADAS3‐71	477	8.8 [4.5–14.0]
C‐HAQ score	601	0.625 [0.125–1.375]
VAS pain, mm	589	30 [8–58]
VAS general evaluation, mm	582	21 [4–50]
IPQ, mean ± SD		
Timeline (range 5–30)	225	17.1 ± 4.2
Consequences (range 0–30)	225	18.4 ± 5.0
Personal control (range 5–25)	225	18.7 ± 4.4
Treatment control (range 5–25)	225	18.6 ± 2.9
Illness coherence (range 5–25)	225	15.7 ± 4.5
Timeline cyclical (range 5–20)	225	12.7 ± 2.9
Emotional representations (range 5–30)	225	18.7 ± 5.4
CHQ		
Physical functioning	353	33 [6–67]
Role/social limitations–emotional/behavioral	351	0 [0–56]
Role/social limitations–physical	352	33 [0–67]
Bodily pain/discomfort	334	60 [30–80]
Behavior	334	28 [15–44]
Mental health	333	25 [15–40]
Self‐esteem	334	29 [13–46]
Parental impact, emotional	354	33 [17–67]
Parental impact, time	352	11 [0–33]
Family activities	354	25 [8–46]
Family cohesion	354	15 [15–40]

Values are the median [interquartile range], unless otherwise indicated. ILAR = International League Against Rheumatism; RF = rheumatoid factor; JADAS‐71 = Juvenile Arthritis Disease Activity Score based on 71 joint count; JADAS3‐71 = JADAS‐71 without erythrocyte sedimentation rate; C‐HAQ = Childhood Health Assessment Questionnaire; VAS = visual analog scale; IPQ = Illness Perception Questionnaire; CHQ = Child Health Questionnaire.

Patients with available C‐HAQ scores, IPQ score, or CHQ‐PF50 scores did not differ from patients without these scores with respect to age, sex, or limited and active joint count. However, median physician global score was significantly higher in those with available data compared to those with missing data: C‐HAQ 30 (95% CI 17, 57) versus 24 (95% CI 13, 45), *P* = 0.0016; IPQ 32 (95% CI 16, 58) versus 24 (95% CI 11, 47), *P* = 0.0028; CHQ‐PF50 31 (95% CI 17, 59) versus 23 (95% CI 10, 42), *P* < 0.001.

### SES and disease subtypes

Based on the national IMD ranking distribution, 176 children (19.1%) were assigned to the high‐SES group, 411 (44.5%) to the middle‐SES group, and 336 (36.4%) to the low‐SES group. At baseline, the percentages of patients according to the ILAR JIA subtypes for the high‐SES, middle‐SES, and low‐SES groups were systemic arthritis (8.0%, 6.4%, and 3.6%, respectively), persistent oligoarthritis (47.5%, 50.7%, and 53.4%, respectively), extended oligoarthritis (2.5%, 2.4%, and 1.6%, respectively), rheumatoid factor–negative polyarthritis (19.1%, 18.8%, and 14.3%, respectively), rheumatoid factor–positive polyarthritis (2.5%, 3.0%, and 4.6%, respectively), enthesitis‐related arthritis (5.6%, 5.9%, and 4.2%, respectively), psoriatic arthritis (6.2%, 4.0%, and 6.2%, respectively), and unclassifiable/other (8.6%, 8.8%, and 12.1%, respectively).

### Association between socioeconomic status and clinical and patient‐reported outcomes

The relationships between socioeconomic status and clinical and patient‐reported outcomes at baseline are shown in Table 2. Age at onset and sex did not differ significantly between the SES groups. Although delay to first rheumatology visit did not differ significantly between the 3 SES groups, more patients in the low‐SES group were referred via the emergency room, general practitioner, or orthopedicsthan by pediatrics compared to patients in the high‐SES group.

**Table 2:** Relationships between socioeconomic status (SES) and clinical and patient‐reported outcomes*

	High‐SES group, reference group (n = 176)	Middle‐SES group (n = 411)	Middle‐ vs. high‐SES group	Low‐SES group (n = 336)	Low‐ vs. high‐SES group
Age at onset, years	6.3 [2.8–10.2]	6.0 [2.5–10.5]	−0.31 (−1.59, 0.97)^a^	7.4 [3.3–11.2]	1.07 (−0.26, 2.39)^a^
Sex, female	109 (62)	261 (64)	0.93 (0.65, 1.35)^b^	211 (63)	0.96 (0.66, 1.40)^b^
Source of referral
Emergency room	3 (1.7)	17 (4.3)	1.01 (−0.25, 2.26)^c^	28 (5.6)	1.96 (0.74, 3.19) sig.^c^
General practitioner	30 (17.1)	62 (15.5)	−0.001 (−0.51, 0.50)^c^	66 (20.2)	0.52 (0.004, 1.03) sig.^c^
Orthopedics	38 (21.7)	113 (28.3)	0.36 (−0.09, 0.81)^c^	105 (32.1)	0.75 (0.28, 1.21) sig.^c^
Pediatrician	87 (49.7)	180 (45)	Comparator	114 (34.9)	Comparator
Other	17 (9.7)	28 (7)	−0.23 (−0.88, 0.43)^c^	14 (4.3)	−0.46 (−1.22, 0.30)^c^
Delay to pediatric rheumatologist, months	5.3 [2.9–12.6]	5.9 [3.1–12.0]	0.56 (−0.42, 1.55)^a^	5.6 [2.6–11.1]	0.27 (−0.75, 1.28)^a^
Active joint count	2 [1–4]	1 [1–5]	0 (−2.53, 2.53)^a^	2 [1–4]	0 (−2.61, 2.61)^a^
Limited joint count	1 [0–3]	1 [1–3]	0 (−2.31, 2.31)^a^	1 [0–3]	0 (−2.38, 2.38)^a^
Physician global assessment, mm	29 [13–53]	29 [15–51]	0 (−7.11, 7.11)^a^	29 [17–51]	0 (−7.32, 7.32)^a^
JADAS‐71	10.2 [5.8–15.8]	10.8 [6–18.1]	0.60 (−2.40, 3.60)^a^	12.2 [5.5–16.7]	2.2 (−0.93, 5.33)^a^
JADAS3‐71	10.2 [5.8–15.8]	10.8 [6–18.1]	−0.30 (−2.39, 1.79)^a^	12.2 [5.5–16.7]	0.80 (−1.39, 2.99)^a^
C‐HAQ score	0.5 [0–0.875]	0.625 [0.125–1.375]	0.37 (0.20, 0.67) sig.^d^0.10 (−0.11, 0.30)^e^	0.875 [0.313–1.625]	0.28 (0.14, 0.55) sig.^d^0.26 (0.05, 0.48) sig.^e^
VAS pain, mm	18.5 [4–48]	30 [6–56]	1.08 (0.51, 2.28)^d^0.20 (0.01, 0.41)^e^	39 [13–63.5]	0.43 (1.16, 1.13)^d^0.29 (0.07, 0.51) sig.^e^
VAS general evaluation, mm	15 [4–35]	20 [3–50]	1.54 (0.56, 4.20)^d^0.30 (0.06, 0.54) sig.^e^	30 [6–52]	0.46 (0.11, 1.86)^d^0.39 (0.14, 0.63) sig.^e^
IPQ					
Timeline	15.7 (3.6)	17.5 (3.4)	1.73 (0.31, 3.14) sig.	17.5 (5.1)	1.75 (0.28, 3.22) sig.
Consequences	16.9 (4.8)	18.6 (4.8)	1.68 (0.00, 3.37)	19.2 (5.1)	2.32 (0.58, 4.07) sig.
Personal control	18.6 (4.0)	18.5 (3.7)	−0.13 (−1.63, 1.38)	18.9 (5.2)	0.22 (−1.34, 1.78)
Treatment control items	19.9 (1.9)	18.1 (2.9)	−1.76 (−2.74, −0.77) sig.	18.5 (3.3)	−1.38 (−2.40, −0.36) sig.
Illness coherence items	17.0 (3.4)	15.7 (4.5)	−1.24 (−2.76, 0.28)	15.0 (4.8)	−2.0 (−3.59, −0.45) sig.
Timeline cyclical	12.4 (2.6)	12.6 (2.9)	0.19 (−0.81, 1.20)	12.9 (3.1)	0.50 (−0.54, 1.54)
Emotional representations	17.9 (4.2)	19.0 (5.8)	1.06 (−0.79, 2.91)	18.7 (5.5)	0.77 (−1.15, 2.69)
CHQ					
Physical functioning	11.1 [0–44]	33 [11–67]	0.43 (0.23, 0.81) sig.^d^0.20 (−0.01, 0.43)^e^	44 [11–72]	0.38 (0.19, 0.75) sig.^d^0.36 (0.11, 0.60) sig.^e^
Role/social limitations–emotional/behavioral	0 [0–22]	0 [0–56]	0.87 (0.50, 1.52)^d^0.31 (0.06, 0.56) sig.^e^	22 [0–67]	0.66 (0.37, 1.18)0.42 (0.16, 0.69) sig.^e^
Role/social limitations–physical	17 [0–33]	33 [0–67]	16.67 (−26.63, 59.96)^a^	50 [0–67]	33.33 (−12.36, 79.03)^a^
Bodily pain/discomfort	50 [20–70]	60 [40–80]	10 (−16.30, 36.30)^a^	60 [40–80]	10 (−16.63, 36.63)^a^
Behavior	23 [14–32]	32 [19–44]	8.33 (2.79, 13.89) sig.^a^	25 [15–44]	1.67 (−4.15, 7.48)^a^
Mental health	23 [15–35]	25 [15–40]	0 (−13.0, 13.0)^a^	25 [15–45]	0 (−13.4, 13.4)^a^
Self‐esteem	25 [15–33]	29 [17–50]	0.49 (0.15, 1.61)^d^0.15 (−0.04, 0.34)^e^	25 [13–45]	1.92 (0.72, 5.17)^d^0.14 (−0.06, 0.34)^e^
Parental impact, emotional	33 [17–50]	42 [17–67]	8.33 (−14.09, 30.75)^a^	33 [17–67]	3.27−e14 (−23.29, 23.29)
Parental impact, time	11 [0–33]	11 [0–44]	0.996 (0.57, 1.74)^d^0.34 (0.08, 0.59) sig.^e^	11 [0–44]	0.71 (0.40, 1.29)^d^0.24 (−0.02, 0.49)^e^
Family activities	17 [4–38]	25 [8–50]	0.79 (0.39, 1.59)^d^0.24 (0.01, 0.47) sig.^e^	29 [8–50]	0.58 (0.27, 1.25)^d^0.24 (0.01, 0.48) sig.^e^
Family cohesion	15 [15–40]	15 [15–40]	0 (−40.64, 40.64)^a^	15 [15–40]	0 (−42.65, 42.65)^a^

Values for high‐, middle‐, and low‐SES groups are median [interquartile range] for continuous variables and number (percentage) for categorical variables. Comparison columns include values for β‐coefficent/odds ratio/rate ratio (95% confidence interval). Unless otherwise indicated by a footnote, values are from β‐coefficient linear regression analysis. Sig. = statistically significant at *P <* 0.05; JADAS‐71 = Juvenile Arthritis Disease Activity Score based on 71 joint count; JADAS3‐71 = JADAS‐71 without erythrocyte sedimentation rate; C‐HAQ = Childhood Health Assessment Questionnaire; VAS = visual analog scale; IPQ = Illness Perception Questionnaire; CHQ = Child Health Questionnaire.

a β‐coefficient median regression model.

b Odds ratio logistic regression model.

c Rate ratio multinominal logistic model.

d Odds ratio zero‐inflated part of ZINB model.

e β‐coefficient count part of ZINB model.

No significant differences in median physician‐reported outcome scores were observed, including active joint count, limited joint count, and physician global assessment. In addition, both JADAS‐71 and JADAS3‐71 did not differ significantly between the SES groups. Compared to children in the high‐SES group, those in the low‐SES group were significantly less likely to have a C‐HAQ score of zero (OR 0.28, [95% CI 0.14, 0.55], *P* < 0.001), and within the negative binominal part, the rate ratios indicate that the mean C‐HAQ score is approximately 30% higher than in the high‐SES group (β 0.26, [95% CI 0.05, 0.48], *P* = 0.015). Compared to the children in the high‐SES group, children in the middle‐SES group were also less likely to have a C‐HAQ score of zero (OR 0.37, [95% CI 0.20, 0.67], *P* = 0.01), but the results in the negative binominal part were not statistically significant. A similar result was found for the physical activity score included in the CHQ‐PF50 questionnaire (Table 2). Although compared to children in the high‐SES group, children in the low‐SES group were not more likely to have excessive zero scores for VAS pain and VAS general evaluation, but those not scoring zero indicated they had more pain (β 0.07, [95% CI 0.07, 0.51], *P* = 0.010) and had a higher general evaluation score (β 0.39, [95% CI 0.14, 0.63], *P* = 0.002).

Compared to the high‐SES group, parents or adolescents in the middle‐SES and low‐SES groups thought that arthritis had large consequences for themselves and their family (β 1.68, [95% CI 0.00, 3.37], *P* = 0.05 for middle‐SES versus high‐SES, and β 2.32, [95% CI 0.58, 4.07], *P* = 0.009 for low‐SES versus high‐SES), and they thought that the arthritis would last a long time (β 1.73, [95% CI 0.31, 3.14], *P* = 0.017 for middle‐SES versus high‐SES, and β 1.75, [95% CI 0.28, 3.22], *P* = 0.020 for low‐SES versus high‐SES) (Table 2). Furthermore, patients in the low‐SES and middle‐SES groups thought that the arthritis treatment would be less effective in curing the arthritis compared to patients in the high‐SES group (β −1.76, [95% CI −2.74, −0.77], *P* = 0.001 for middle‐SES versus high‐SES, and β −1.38, [95% CI −2.40, −0.36], *P* = 0.009 for low‐SES versus high‐SES).

Compared to the high‐SES group, parents of children included in the middle‐SES and the low‐SES group scores more often reported that their children's school work or activities with friends had been limited due to emotional difficulties or problems with their behavior (role/social limitations–emotional/behavioral: β 0.31, [95% CI 0.06, 0.56], *P* = 0.014 for the middle‐SES group, and β 0.42, [95% CI 0.16, 0.69], *P* = 0.001 for the low‐SES group). The same parents also more often reported that their child's health or behavior had an impact on the family's activities (β 0.24, [95% CI 0.01, 0.47], *P* = 0.039 for the middle‐SES group, and β 0.24, [95% CI 0.01, 0.48], *P* = 0.040 for the low‐SES group). When applying Bonferroni's adjustment, resulting in a *P* value of 0.0017, the findings for the C‐HAQ score remained statistically significant.

## DISCUSSION

This is the first study looking at the association between SES using national deprivation indices and disease severity at presentation, delay to pediatric rheumatology care, and illness perception in patients with JIA. Despite similar disease activity scores obtained in the clinic, patients from a low‐SES background rated their general disease activity, pain, and functional disability worse than patients from a high‐SES background. Parents from a low‐SES background also reported that their child with JIA had been limited in their schoolwork or activities with friends due to emotional difficulties, had problems with their behavior and limited family activities, or had caused tension in the family. Part of these findings is similar to those found in previous studies defining SES at an individual level, such as insurance information 17, education level of parents 18, 19, 28, living in a Canadian reserve 29, and distance to health care centers 19. In this study we used area deprivation level data, which conceptually might be slightly different. SES levels measured at an individual level and at an area level have both shown to be independent predictors of arthritis health 12. A disadvantage of using individual‐level data in this study might have been that the data will reflect the parent's SES. Furthermore, data on income and education of parents was missing for a large proportion of our study population. We therefore chose to use SES based on area‐level data because we think they are probably more accurate, including data on income, employment, health deprivation, disability, education, skills, training, barriers to housing and services, crime, and living environment. A disadvantage might be ecological fallacy. The discrepancy between the observed clinical findings and parent/patient‐reported findings in our study may partly be explained by unmeasured confounding such as comorbidities. Population‐based surveys have shown that children from deprived areas are more likely to have other chronic diseases such as asthma and diabetes mellitus 30, 31, 32.

We also found that the expectations in parents of children and young adolescents with arthritis regarding the duration of the disease, consequences of the disease, and the effect of treatment were lower in patients with a low SES compared to those with a high SES. This finding, together with the findings that JIA has a greater impact on a child's schoolwork, activities with friends, and family in families from a low SES, is of importance when discussing the consequences of JIA with the child and their family in the clinic. Advice to the family about how to cope with the disease may therefore differ depending on the SES of the child. A previous study also showed that children with JIA from a low SES are less likely to adhere to their medication 33. The results from our study and the earlier study, therefore, warrant special attention from the pediatric rheumatology multidisciplinary team to provide additional social, psychological, and educational resources and support to those children and families who we would suggest are at greater risk of poor functional outcome. To aid better support, we need to understand which factors contribute to more disability and disease concerns in people with a low SES. Is there a general difference in disease perception between people with low and high SES, or are higher disability scores partly explained by other factors such as chronic comorbidities, as previously mentioned? Providing better background information about the disease and its consequences could help adolescents or adults of children with JIA to better understand the disease. To determine which other factors may contribute to differences in patient‐reported outcome scores, further research is necessary into the sensitivity of these measures and the discriminative ability in low‐ and high‐SES groups over time.

Unfortunately, one of the limitations of our study was the cross‐sectional design, and causation could not be ascertained. However, we did find that patients in the high‐SES group were mainly referred to the pediatric rheumatologist via their pediatricians, whereas patients in the low‐SES group were more often referred via the emergency room, or from other specialities. Interestingly, the delay to the first pediatric rheumatologist appointment did not differ between the 3 SES groups. Because the vast majority of health care provision in the UK is public and free at point of access, time to presentation should therefore not be affected by affordability, but could affect delay in presentation, since first symptoms are based on personal circumstances and/or decisions. In a German study, delayed referral was associated with the primary physician's subspecialty (i.e., orthopedic surgeon) and distance to the pediatric hospital, so that patients living in more remote areas had the longest referral time 19. Notably, more remote areas do not necessarily mean low SES, and it is therefore difficult to compare the results, since we did not calculate the distance between the child's home address and the hospital. To further entangle the relationship between SES and disease activity in JIA, longitudinal data are required, since not only may SES impact disease‐related factors and patient‐reported outcomes, but caring for children with a chronic disease such as JIA may also impact families' cost of living and thus have an effect on SES, in that families may have to pay for travel to hospitals, pay for adaptations and devices, take days off from work, or even stop working to take care of their child.

We were only able to include patients recruited to CAPS from England and not those patients recruited from Scotland, because of the differences in defining deprivation scores in England and Scotland. The postcode of the home address where patients lived at the time of inclusion in CAPS was used to determine the deprivation status. Possibly patients had not been living very long at that address. However, even if they moved homes in the year prior to inclusion, the SES is unlikely to have changed very much. We sought to determine the association between SES and quite a large number of demographic, clinical, and patient‐reported outcomes (n = 30). Some findings may have been significant by chance, although some variables remained significant after adjusting for multiple tests.

In conclusion, the way parents and children perceive the child's illness and the consequences of the disease differs between SES groups. Patients from a low‐SES background score their disease activity and functional disability higher than patients from a high‐SES background, whereas no differences were found in disease activity scores obtained in the clinic. This study suggests that it is important to take SES into account when patients with JIA arrive at the clinic for the first time.

## AUTHOR CONTRIBUTIONS

All authors were involved in drafting the article or revising it critically for important intellectual content, and all authors approved the final version to be submitted for publication. Dr. Verstappen had full access to all of the data in the study and takes responsibility for the integrity of the data and the accuracy of the data analysis.

**Study conception and design.** Verstappen, Cobb, Foster, Baildam, Wedderburn, Hyrich, Thomson.

**Acquisition of data.** Foster, Baildam, Wedderburn, Davidson, Ioannou, Chieng, Thomson.

**Analysis and interpretation of data.** Verstappen, Fu, Hyrich, Thomson.
